# Neurotropic virus infection and neurodegenerative diseases: Potential roles of autophagy pathway

**DOI:** 10.1111/cns.14548

**Published:** 2023-12-11

**Authors:** Yu‐jia Zhao, Kai‐fei Xu, Fu‐xing Shu, Feng Zhang

**Affiliations:** ^1^ Laboratory Animal Centre Zunyi Medical University Zunyi Guizhou China; ^2^ Key Laboratory of Basic Pharmacology of Ministry of Education and Joint International Research Laboratory of Ethnomedicine of Ministry of Education and Key Laboratory of Basic Pharmacology of Guizhou Province Zunyi Medical University Zunyi Guizhou China; ^3^ Bioresource Institute for Healthy Utilization Zunyi Medical University Zunyi Guizhou China

**Keywords:** Alzheimer's disease, autophagy, neurodegenerative diseases, neurotropic virus, Parkinson's disease

## Abstract

Neurodegenerative diseases (NDs) constitute a group of disorders characterized by the progressive deterioration of nervous system functionality. Currently, the precise etiological factors responsible for NDs remain incompletely elucidated, although it is probable that a combination of aging, genetic predisposition, and environmental stressors participate in this process. Accumulating evidence indicates that viral infections, especially neurotropic viruses, can contribute to the onset and progression of NDs. In this review, emerging evidence supporting the association between viral infection and NDs is summarized, and how the autophagy pathway mediated by viral infection can cause pathological aggregation of cellular proteins associated with various NDs is discussed. Furthermore, autophagy‐related genes (ARGs) involved in Herpes simplex virus (HSV‐1) infection and NDs are analyzed, and whether these genes could link HSV‐1 infection to NDs is discussed. Elucidating the mechanisms underlying NDs is critical for developing targeted therapeutic approaches that prevent the onset and slow the progression of NDs.

## Graphical abstract

Virus infection, autophagy, and neurodegenerative diseases.

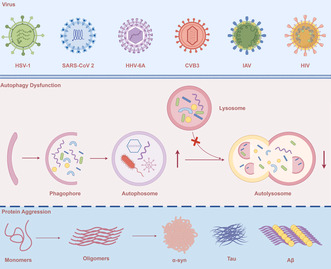

## 1 INTRODUCTION

Neurodegenerative diseases (NDs) are devastating neurological diseases affecting the central nervous system (CNS) and are characterized by the progressive degeneration and loss of neurons in various regions of the nervous system.^1^ Pathologically, the majority of NDs are associated with the accumulation and aggregation of cellular proteins.^2^, ^3^ Notably, α‐synuclein (α‐syn) aggregates are observed in Parkinson's disease (PD), multiple systems atrophy (MSA), and dementia with Lewy bodies.^4^ Also, extracellular amyloid‐β (Aβ) plaques and intraneuronal tangles of hyperphosphorylated tau are formed in the brain of people with Alzheimer's disease (AD),^5^ and TAR DNA‐binding protein 43 (TDP‐43) or fused in sarcoma (FUS) aggregates are found in patients with Amyotrophic Lateral Sclerosis (ALS).^6^, ^7^ These pathological proteins possess the capacity to propagate in a manner akin to prions, aggregating and forming pathogenic plaques, ultimately leading to NDs.^8^, ^9^, ^10^ An imbalance in cellular processes involving protein homeostasis, which is the generation of misfolded proteins and their degradation, plays a critical role in these processes.^11^ Viral infections can profoundly disrupt protein homeostasis and increase cells susceptibility to protein misfolding.^12^ Additionally, viral cellular responses, such as the release of pro‐inflammatory cytokines and chemokines, can contribute to protein homeostasis.^13^ Therefore, viruses, particularly neurotropic viruses, are suspected to be etiological factors in various NDs.

Neurotropic viruses, constituting a class of emerging and re‐emerging pathogens, exhibit a specific affinity for CNS and disrupt the integrity of CNS; these viruses include Japanese encephalitis virus (JEV) in *Flaviviridae*, Herpes simplex virus type 1 (HSV‐1) in *Herpesviridae*, Influenza A virus (IAV) in *Orthomyxoviridae*, and Coxsackievirus B3 (CVB3) in *Picornaviridae*.^14^, ^15^ They can enter the CNS through various distinct strategies, leading to a range of neurological symptoms.^16^ One strategy utilized by viruses of significant importance involves infiltration into the peripheral nervous system and subsequent transportation via axon fibers into the CNS.^17^ Neurotropic viruses, in addition to exploiting the peripheral nervous system, employ various other approaches to circumvent host barrier systems and directly access the CNS. For instance, Zika virus (ZIKV), human cytomegalovirus (HCMV), and human immunodeficiency virus (HIV) can enter the CNS via a “Trojan horse” mechanism, whereby they infect immune cells, such as macrophages, monocytes, and dendritic cells that function as carriers to traffic the virus into the CNS.^16^, ^18^, ^19^ Furthermore, viral infections have been demonstrated to stimulate the production of pro‐inflammatory cytokines and chemokines, including IL‐6, IL‐8, TNF‐α, CCL2, and CCL5, which can trigger a cytokine storm.^16^, ^20^ In addition, viral infections are associated with a reduction in the expression of tight junction proteins, such as claudin‐1, claudin‐5, occludin, and ZO‐1.^21^, ^22^, ^23^ These alterations possess the capacity to augment vascular permeability and permit viruses to breach this barrier system and enter the CNS. Neurotropic viruses are likely to utilize one or multiple entry routes to gain access to the CNS. Once entering the CNS, viruses can trigger the activation of microglia and astrocytes,^24^, ^25^ neuroinflammation,^25^ immune responses,^14^ oxidative stress,^26^ protein aggregation,^27^ and disruption of gut microbial balance.^28^, ^29^ These factors can initiate and exacerbate the onset and progression of NDs.^30^

Autophagy, a vital cellular process responsible for delivering cellular contents, including damaged organelles, protein aggregates, and invading pathogens, to lysosomes for degradation, plays a crucial role in maintaining cellular homeostasis.^31^, ^32^, ^33^ Nevertheless, both the capacity and efficiency of autophagy decline during aging and upon viral infection.^34^ Previous studies demonstrated that autophagy deficits were implicated in ND pathogenesis.^35^, ^36^, ^37^ These deficits not only cause protein aggregation and phosphorylation but also potentially enhance glial activation, neuroinflammation, and neuronal cell death.^38^, ^39^, ^40^ Moreover, α‐syn shows the ability to impede autophagy,^41^ whereas Aβ, or phosphorylated Tau, can trigger defective autophagy and mitophagy.^42^ These findings suggest a potential correlation between the autophagy pathway and NDs. Noteworthy, viruses possess the capacity to directly or indirectly manipulate the autophagy process for their own benefit during infection. Regarding the association of the autophagy process mediated by viral infection with NDs, numerous studies have indicated that viruses could hinder the formation of autophagosomes or the fusion of autophagosomes and autolysosomes, thereby leading to protein misfolding, aggregation, and subsequent dissemination.^43^ These findings indicate that autophagy may serve as a novel mechanism linking viral infection to NDs.

In this review, we summarized the emerging evidence supporting an association between viral infections and NDs and discussed the contribution of autophagy, a cellular process that was modulated by viral infections, to the pathogenesis of NDs. Furthermore, we analyzed the involvement of autophagy‐related genes (ARGs) in both HSV‐1 infection and NDs and explored whether these genes could link HSV‐1 infection to NDs.

## 2 VIRAL INFECTION IS A RISK FACTOR IN NEURODEGENERATIVE DISEASES

**FIGURE 1:**
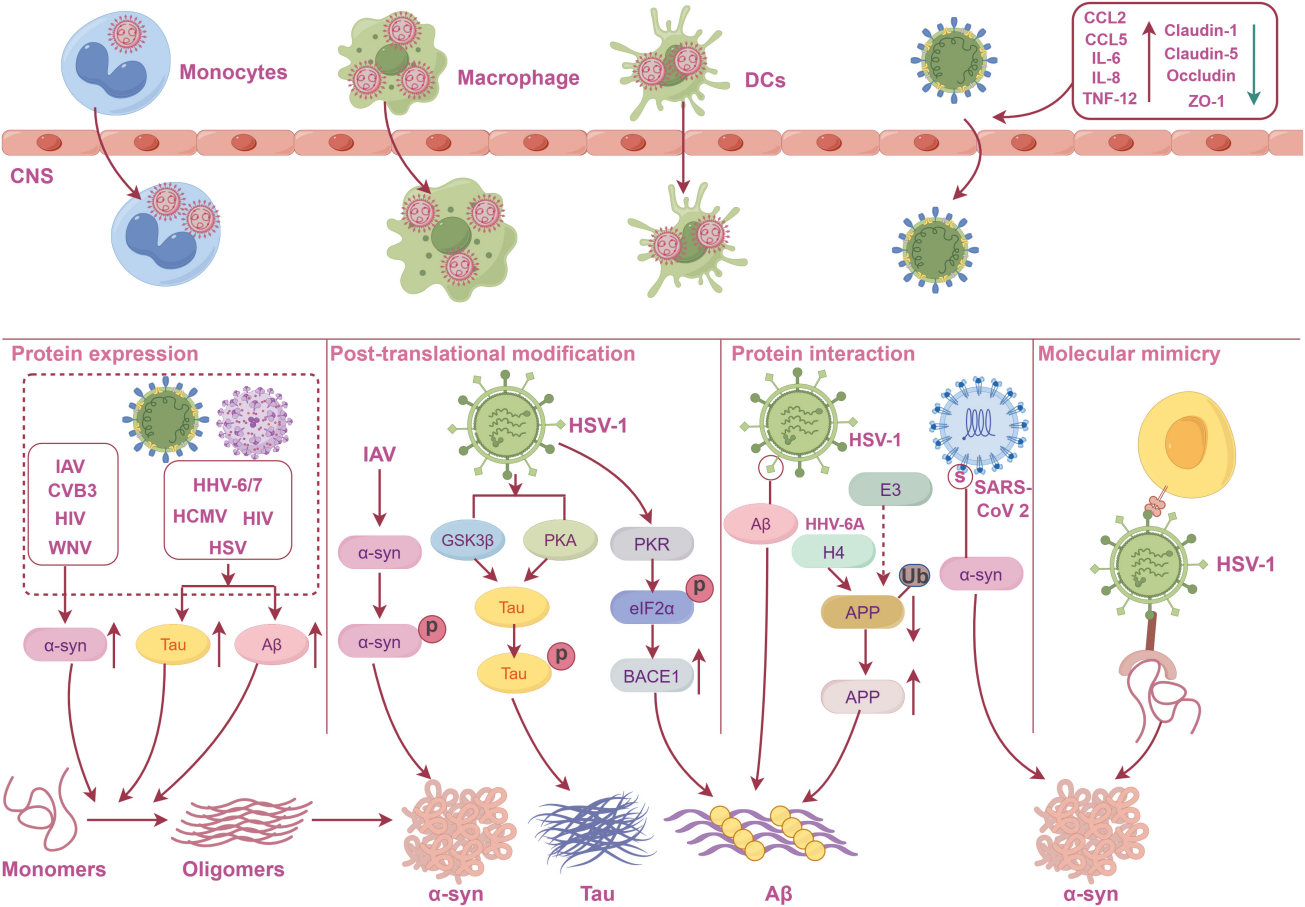
Potential mechanisms of viral infections in ND pathogenesis. Neurotropic viruses can enter the CNS via a “Trojan horse” mechanism or altered vascular permeability. Once in the CNS, virus infection enhances the expression of proteins associated with NDs, which can reduce the nucleation barrier for protein aggregation. HSV‐1 infection activates GSK3β, PKA, and PKR. Among them, GSK3β and PKA are responsible for Tau phosphorylation, while PKR catalyzes eIF2α phosphorylation, leading to BACE1 translation and Aβ accumulation. Viral surfaces or specific viral proteins and antibodies can interact with proteins associated with NDs, ultimately leading to their deposition. BACE1, β‐site amyloid precursor protein cleaving enzyme 1; CNS, central nervous system; eIF2α, elongation initiation factor 2α; GSK3β, glycogen synthase kinase 3β; HSV‐1, Herpes simplex virus type 1; NDs, neurodegenerative diseases; PKA, protein kinase A; PKR, RNA‐activated protein kinase.

Viruses, especially those with neurotropic properties, are increasingly recognized as potential risk factors that contribute to the onset and progression of numerous NDs.^44^ Mounting evidence supports the potential associations between viral infections and an elevated risk of NDs (Table 1). For instance, CVB3 infection has been verified to be related to PD,^39^ while HSV‐1 with PD, AD, Multiple sclerosis (MS), and ALS,^45^, ^46^, ^47^, ^48^ Enteroviruses (EVs) with ALS,^49^ and Epstein‐Barr virus (EBV) with MS.^50^ Recently, available datasets from two extensive population‐based studies, termed FinnGen and UK Biobank, were leveraged to investigate risk factors. In FinnGen, 45 notable connections between viral infections and NDs were discovered, with 22 of these associations being replicated in the UK Biobank. Notably, the most robust hazard ratio was observed for the association between viral encephalitis and AD.^51^ Overall, this is the first study to systematically investigate the association between various viral pathogens and different NDs, including PD, AD, ALS, MS, generalized dementia (DEM), and vascular dementia (VAS).^45^, ^51^ Furthermore, through virome analysis, nine viruses were identified to be present in various brain tissues of the CNS in individuals with PD, and the positive rates of viruses were higher in PD patients than those in control group patients.^52^ Strikingly, recent studies provide evidence in support of the notion that the administration of vaccines or antiviral agents may mitigate the risk of NDs in patients with viral infection.^53^, ^54^, ^55^ Taken together, these collective findings support the hypothesis that viral infections contribute to an increased risk of developing NDs, and the potential mechanisms of viral infections in ND pathogenesis are shown in Figure 1.

**TABLE 1:** Overview of neurotropic viruses associated with NDs.

NDs	Neurotropic viruses	References
PD	CVB3, DENV, EBV, HCMV, HSV‐1, HHV‐5/6, HIV, IAV, JEV, VZV, WEEV, WNV	46, 72, 105, 163, 164
AD	CMV, EBV, HCMV, HHV‐6/7, HIV, HSV‐1/2, HLTV‐1, IAV, HERV, VZV, ZIKV	51, 164, 165, 166, 167
MS	EBV, HSV‐1, HHV‐6, HIV, VZV, WNV	48, 50, 164, 168, 169
ALS	EVs, HERV, HIV, HLTV‐1, HSV‐1, TMEV	49, 66, 164, 170
HD	HIV	171

As a risk factor, viral infection enhances the expression of proteins associated with NDs, which can reduce the nucleation barrier for protein aggregates and result in amyloid formation.^40^ For instance, α‐syn expression is elevated following infection with CVB3, IAV, West Nile virus (WNV), and HIV,^39^, ^56^ while TDP‐43 and FUS levels increase upon HSV‐1 and Theiler's mouse encephalomyelitis virus (TMEV) infection.^56^ Furthermore, HSV, HIV, HCMV, and HHV‐6/7 infections result in elevated Aβ and Tau levels.^56^, ^57^ In addition, investigations revealed that these proteins possessed antiviral properties, effectively inhibiting viral infection.^58^, ^59^ Conversely, conflicting studies cast doubt on the antiviral function of Aβ, suggesting that it does not impede the spread of viruses to the CNS upon infection.^60^, ^61^

Post‐translational modifications are well‐characterized cellular mechanisms of protein function regulation, and phosphorylation is the most extensively explored post‐translational modification.^62^ HSV‐1 infection activates glycogen synthase kinase 3β (GSK3β) and protein kinase A (PKA), which are responsible for Tau phosphorylation at various sites, including serine 202, threonine 212, serine 214, serine 396, and serine 404, leading to the hyperphosphorylation of Tau and loss of neurons in primary cell culture.^63^ Moreover, HSV‐1 could activate RNA‐activated protein kinase (PKR), an enzyme that catalyzes elongation initiation factor 2α (eIF2α) phosphorylation, thereby leading to β‐site amyloid precursor protein cleaving enzyme 1 (BACE1) translation and Aβ accumulation.^64^ Also, infection with viruses, such as IAV H5N1,^65^ can cause α‐syn phosphorylation and aggregation.

Viral surfaces or specific viral proteins can interact with proteins associated with NDs, thereby directly influencing their solubility and stability, ultimately leading to their deposition.^66^ For example, HSV‐1, HHV‐6A, and HHV‐6B surface glycoproteins can directly bind to Aβ oligomers, thus accelerating Aβ deposition in mice and in 3D cultures of human neurons.^58^ Additionally, HHV‐6A U4 protein competes with amyloid precursor protein (APP) for binding E3 ubiquitin ligase, resulting in the inhibition of APP ubiquitin modification and clearance.^67^ Consequently, this outcome leads to an elevation in APP expression and Aβ deposition, which are recognized as the characteristic hallmarks of AD.^67^ In 2019, the outbreak of the severe acute respiratory syndrome coronavirus 2 (SARS‐CoV‐2) infection damaged multiple organ systems, including the brain.^68^ The interaction of the SARS‐CoV‐2 spike or nucleocapsid protein with α‐syn accelerates its aggregation, leading to the development of a Lewy body‐like pathology.^69^, ^70^ Moreover, binding of SARS‐CoV‐2 spike protein and heparin expedites the aggregation of Aβ, α‐syn, tau, and TDP‐43, ultimately causing neurodegeneration in the brain.^71^

Molecular mimicry is characterized by a structural resemblance between viral and host antigens, leading to the activation of T or B‐cell responses that target both host and auto‐antigens.^72^ Interestingly, the U24 membrane protein, encoded by HHV‐6, exhibits specific amino acid similarities with myelin basic protein (MBP), a crucial constituent of the myelin sheath. This shared sequence has the potential to elicit cross‐reactivity between HHV‐6A and MBP.^73^ Moreover, it has been observed that a substantial proportion of T cells that recognize MBP exhibit cross‐reactivity and can be activated by a synthetic peptide corresponding to specific residues of HHV‐6 or EBV in individuals with MS.^74^ Additionally, autoantibodies that recognize HSV‐1 or EBV have been discovered to potentially interact with α‐syn.^75^, ^76^ These findings suggest that viral infections may play an important role in ND pathogenesis through molecular mimicry.

## 3 VIRAL INFECTION MEDIATES THE AUTOPHAGY PROCESS

**FIGURE 2:**
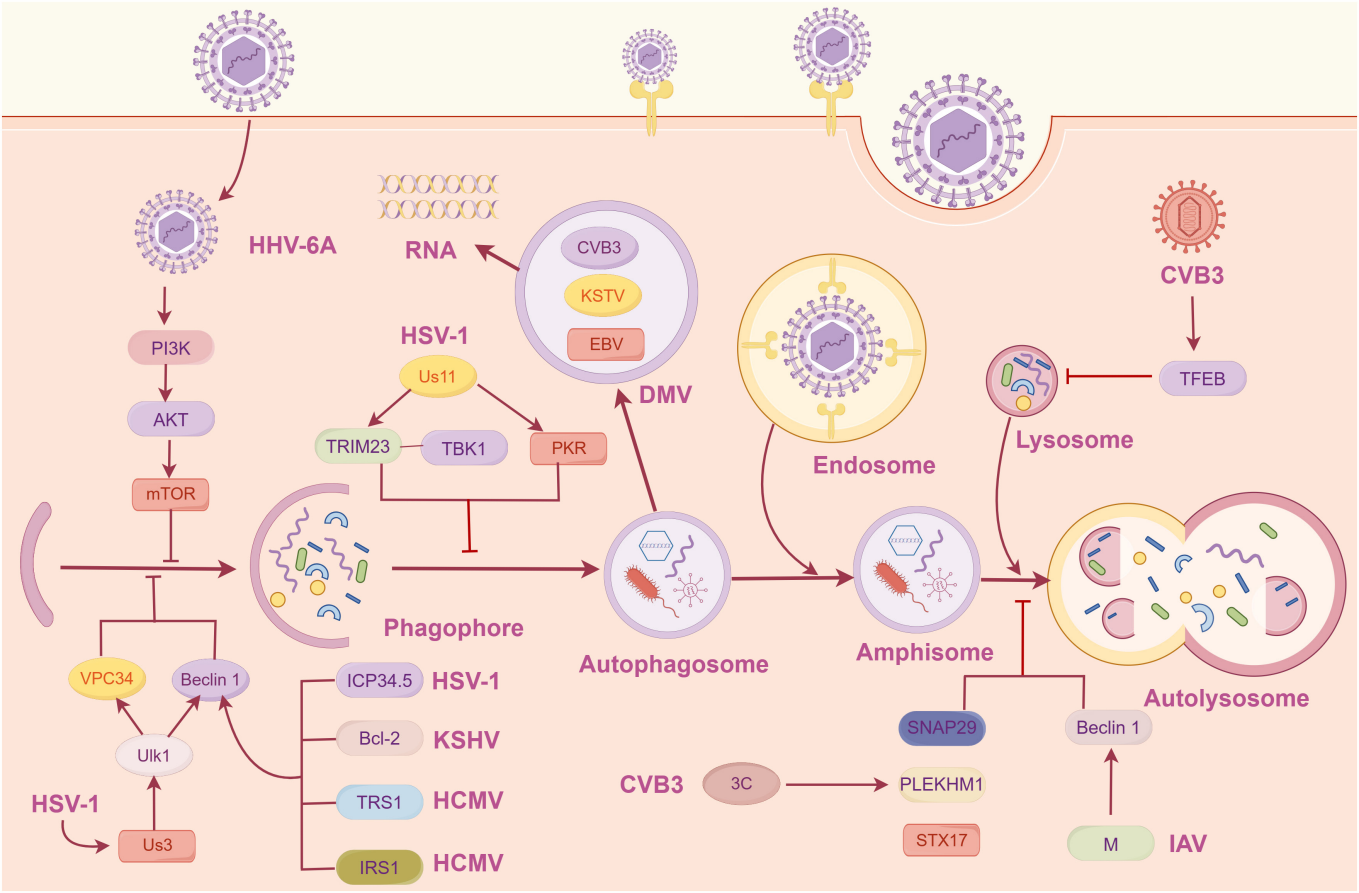
Virus infection mediates the autophagy process. Viral infection inhibits autophagy initiation by activation of the type I PI3K‐AKT–mTOR pathway or the interaction of viral proteins, ICP34.5, Bcl‐2, TRS1, and IRS1, with Beclin 1; HSV‐1 Us3 targets Ulk1 also to hinder autophagy initiation by the class III PI3K‐VPS34‐Beclin 1 pathway; HSV‐1 hinders autophagy by targeting PKR or TRIM23‐TBK1 complex; CVB3, EBV, and KSHV exploit autophagosomes for viral replication; CVB3 proteinase 3C targets host proteins, SNAP29, PLEKHM1, STX17, and TFEB, to inhibit autophagy; IAV M2 impedes autophagosome‐lysosome fusion. CVB3, Coxsackievirus B3; EBV, Epstein‐Barr virus; HSV‐1, Herpes simplex virus type 1; IAV, Influenza A virus; KSHV, Kaposi's sarcoma‐associated herpesvirus; PLEKHM1, pleckstrin homology domain containing protein family member 1; SNAP29, synaptosome associated protein 29; STX17, syntaxin 17; TFEB, transcription factor EB.

Autophagy is a critical mechanism in host defense responses against viral infections.^14^ However, viruses have evolved unique mechanisms to evade these host defense responses by subverting the autophagy process and further interfering with host antiviral signaling triggered by viral infection.^77^, ^78^ Upon viral infection, viruses can use autophagosomes as replication sites, while several specific pathogens inhibit the fusion of autophagosomes and lysosomes and/or inhibit lysosomal degradation by manipulating various signaling pathways (Figure 2). Previous studies have demonstrated that autophagy deficits are implicated in NDs pathogenesis.^35^, ^36^, ^37^ Therefore, we focus on the interaction between autophagy and viruses, discussing how viruses, especially neurotropic viruses, inhibit the autophagy process following infection.

### 3.1 Viral infection inhibits autophagy initiation

Autophagy initiation commences with the formation of phagophores, a process that is regulated by autophagy‐related proteins that are recruited to the membranes of various organelles.^77^ However, viral infections have been observed to hinder autophagy during the initiation phase.^79^, ^80^ This hindrance is accomplished either through the activation of the type I PI3K‐AKT–mTOR pathway or the inhibition of the class III PI3K‐VPS34‐Beclin 1 pathway.^81^ For example, HSV‐1 encodes the neurotoxicity protein ICP34.5, which has been documented to interact directly with Beclin 1, resulting in the inhibition of autophagy and contributing to its pathogenic effects.^79^ Interestingly, an HSV‐1 recombinant virus harboring a mutation in ICP34.5 exhibits an inability to suppress autophagy in neurons. However, deletion of PKR in vivo restores the ability to inhibit autophagy in this context.^79^ HSV‐1 protein Us3 also hinders autophagy initiation by targeting the autophagy‐initiating kinase Ulk1 and Beclin1.^80^ In addition, Bcl‐2, which is encoded by Kaposi's sarcoma‐associated herpesvirus (KSHV), exhibits strong binding affinity for Beclin 1, leading to the inhibition of autophagy.^82^ This inhibitory effect is partially achieved by obstructing the interaction between Beclin 1 and VPS34, thereby reducing the activity of Beclin‐1‐associated class III PI3K.^82^ HCMV proteins, namely TRS1 and IRS1, have been shown to interact with Beclin 1, both of which impede the formation of autophagosomes.^83^

### 3.2 Viral infection suppresses autophagosome‐lysosome fusion

Autophagosome‐lysosome fusion is a crucial event in the autophagy process. Several viral infections, such as IAV,^84^ SARS‐CoV‐2,^85^ and CVB3,^39^ have been shown to inhibit this step and thus facilitate their own replication. In the case of IAV infection, the accumulation of autophagosomes occurs due to the inhibition of their fusion with lysosomes mediated by the viral M2 protein, which possesses proton channel activity.^84^ Increasing evidence shows that the proton channel activity of M2 participates in impeding autophagosome‐lysosome fusion, in contrast to previous findings.^84^, ^86^ Interaction between M2 and Beclin 1 potentially obstruct the fusion process.^84^

Coxsackievirus CVB3, infection has been reported to induce autophagosome formation without promoting protein degradation via lysosomes, indicating that this virus exploits the autophagic machinery to facilitate its replication on the surface of autophagosomes.^87^ Disruption of the class III PI3K signaling complex, which is necessary for the formation of autophagosomes, by Beclin‐1 and VPS34 knockdown leads to a significant reduction in CVB3 infection.^87^ CVB3 specifically targets host proteins synaptosome‐associated protein 29 (SNAP29), pleckstrin homology domain containing protein family member 1 (PLEKHM1), and syntaxin 17 (STX17), which are essential for the regulation of autophagosome fusion, and CVB3 proteinase 3C is responsible for cleaving them in a manner dependent on its own catalytic activity. As a result, the autophagy process is ultimately impaired.^88^, ^89^ Additionally, transcription factor EB (TFEB), a master regulator of autophagy and lysosome biogenesis, has been identified as a novel target of CVB3 proteinase 3C.^90^

### 3.3 Viral infection suppresses lysosomal degradation

Autophagosomes fuse with lysosomes to form autolysosomes, which degrade the contents enclosed in autolysosomes through the action of lysosomal proteases.^91^ Viruses can impede this process to avoid degradation and facilitate their own replication.^78^ For instance, human herpesviruses (HHVs), such as EBV and KSHV, manipulate autophagy and exploit autophagosomes for intracellular transportation, obstructing the final stages to evade degradation in lysosomes.^92^ Similarly, HSV‐1 hinders autophagy by targeting PKR, leading to a blockade of autophagy characterized by a significant reduction in the number of both autophagosomes and autolysosomes.^93^, ^94^ Moreover, the HSV‐1 Us11 protein disrupts the TRIM23‐TBK1 complex spatially, thereby suppressing autophagy.^95^ HHV‐6A inhibits autophagy by activating mTOR, a negative regulator of autophagy.^96^, ^97^ Also, HHV‐6B infection obstructs autophagy in various types of immune cells, including T cells, monocytes, and dendritic cells.^98^, ^99^ This virus induced a selective activation of the unfolded protein response (UPR) to response autophagy, as evidenced by the increased phosphorylation of inositol‐requiring enzyme 1α (IRE1α) and eIF2α, as well as the up‐regulation of activating transcription factor 4 (ATF4) and C/EBP homologous protein (CHOP).^98^

Autophagy and apoptosis are two major interconnected host cell responses to viral infection.^100^ Deficient autophagic flux renders cells more susceptible to apoptosis upon viral infection.^84^ In the case of IAV H1N1 infection, autophagic flux is suppressed, leading to heightened apoptosis, which ultimately contributes to the death of host cells and tissue impairment.^101^ An analysis of gene expression revealed that ARGs involved in autophagosome formation are up‐regulated, while the proteins that facilitate autophagosome‐lysosomal fusion are downregulated in H1N1‐infected cells.^101^

## 4 VIRAL INFECTION, AUTOPHAGY MACHINERY AND NEURODEGENERATIVE DIEASES

**FIGURE 3:**
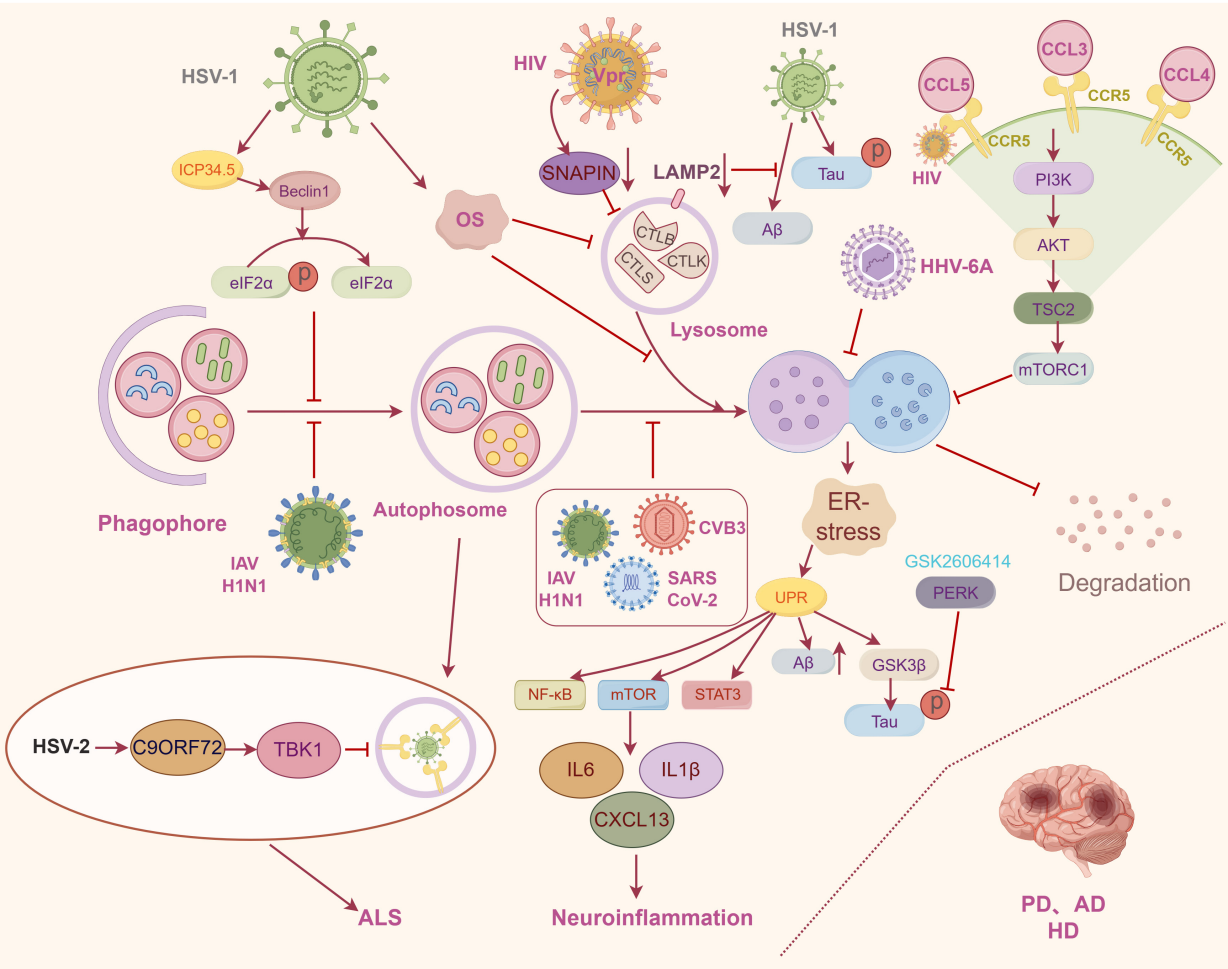
Potential mechanisms of autophagy pathway mediated by virus infection in NDs pathogenesis. HSV‐1 ICP34.5 interacts with Beclin 1 to hinder autophagosome formation by promoting the dephosphorylation of eIF2α; IAV H1N1, CVB3, and SARS‐CoV 2 can inhibit autophagy; oxidative stress induced by HSV‐1 triggers autophagosomes accumulation, inefficient of autophagosome‐lysosome fusion, inhibition of cathepsin activity; HIV Vpr affects SNAPIN expression that is involved in lysosomal maturation; CCL3, CCL4, and CCL5 bind and activate HIV receptor CCR5, which in turn promotes mTORC1 activation by the PI3K‐AKT‐TSC2 pathway and inhibits autophagy; autophagy dysfunction prevents the degradation of α‐syn, Aβ, tau, and HTT, ultimately contributing to NDs pathology. Autophagy dysfunction activates ER stress and triggers the UPR that can activate GSK‐3β to phosphorylate tau protein; PERK inhibitor GSK2606414 reduce tau phosphorylation following HHV‐6A infection. Autophagy reduction and UPR activation caused by HHV‐6A infection is related to the activation of pro‐inflammatory pathways, including the STAT3, NF‐kB and mTOR pathways, leading to the release of inflammatory cytokines such as IL‐6, IL‐1β, and CXCL13, further promoting neuroinflammation; HSV‐2 infection reduces the C9ORF72 level that is responsible for triggering TBK1 phosphorylation and aggregation to induce TDP‐43 aggregation. C9ORF72, chromosome 9 open reading frame 72; CVB3, Coxsackievirus B3; ER, endoplasmic reticulum; GSK3β, glycogen synthase kinase 3β; HHV‐6A, Human herpesvirus 6 A; HIV, Human immunodeficiency virus; HSV‐1, Herpes simplex virus type 1; IAV, Influenza A virus; NDs, neurodegenerative diseases; PERK, protein kinase R‐like endoplasmic reticulum kinase; SARS‐CoV‐2, Severe acute respiratory syndrome coronavirus 2; TDP‐43, TAR DNA‐binding protein 43; UPR, unfolded protein response.

Autophagy is closely involved in the elimination of proteins associated with NDs, and dysfunction within this pathway has been linked to protein accumulation.^102^ Notably, protein aggregates impair autophagy,^41^, ^103^ creating a cycle wherein autophagy is inhibited and protein aggregation is enhanced. Viral infections inhibit autophagy and contribute to ND pathogenesis.^45^, ^81^ Here, we discussed the impact of impaired autophagy mediated by viral infections on ND pathogenies (Figure 3).

### 4.1 Parkinson's disease

Neurotropic viruses, such as IAV,^65^ CVB3,^39^ WNV, Western equine encephalitis virus (WEEV),^104^, ^105^ and JEV, have been shown to induce the formation of α‐syn aggregation in vitro and in vivo following infection, which might trigger PD. Recently, studies have confirmed that viruses can manipulate the autophagy process to promote α‐syn aggregation. For instance, CVB3 infection leads to the emergence of autophagy‐related structures in neurons, which colocalizes with LC3 and pSer129 α‐syn.^39^ Subsequent investigations have revealed that CVB3 infection impedes the later phase of the autophagic process, thereby facilitating the development of Lewy body‐like inclusions containing α‐syn fibrils. Moreover, α‐syn overexpression enhances autophagic flux and accelerates the formation of autophagosomes, which favors CVB3 infection.^39^ IAV H1N1 infection can result in a reduction in the number of available autophagosomes and the hindrance of autophagosome‐lysosome fusion, indicating that it can inhibit the autophagy process at its early and later stages.^55^ Notably, α‐syn inhibits autophagy.^41^ In summary, these effects create a detrimental circle of increased accumulation of misfolded α‐syn in dopaminergic neurons and autophagy, thereby leading to the subsequent loss of dopaminergic neurons.^55^, ^106^ Furthermore, the emerging human coronavirus, SARS‐CoV‐2, has the capacity to invade the brain.^85^ Its accessory protein, open reading frame 3 (ORF3), can block autophagic flux, possibly by inhibiting the fusion of autophagosomes with lysosomes, leading to the accumulation of autophagosomes.^107^, ^108^ The dysfunction of the autophagy‐lysosomal pathway further causes α‐syn aggregation.^108^

The degradation of cytoplasmic material through the autophagy pathway requires an acidic lysosomal environment.^109^ The low pH of the lysosomes triggers conformational changes in proteins, rendering them more vulnerable to proteolytic degradation.^109^ HIV viral protein R (Vpr) causes an obvious increase in the lysosomal pH, impairs lysosome acidification, and consequently impedes the degradative capability of lysosomes, resulting in the accumulation of α‐syn protein.^110^ Moreover, HIV Vpr shows the ability to disrupt lysosome positioning, and the expression of the SNAPIN protein, which is involved in lysosomal maturation, can partially restore lysosomal positioning but does fully alleviate the acidification impairment caused by HIV‐1 Vpr.^110^

### 4.2 Alzheimer's disease

HHVs infections have been implicated in the pathogenesis of AD. These viruses include mainly HHV‐6A/B^111^, ^112^ and HSV‐1,^113^, ^114^ both of which are neurotropic viruses that can cause CNS diseases with neuroinvasive and neurovirulence properties. Recently, studies have linked HHVs infection to the neuropathological hallmarks of AD, proposing that viral infections could directly or indirectly contribute to the accumulation and aggregation of Aβ, and hyperphosphorylation of tau protein, the main component of neurofibrillary tangles.^67^, ^115^, ^116^, ^117^ Regarding the association of autophagy mediated by HHVs infections with AD pathogenesis, it has been reported that viral infections can disrupt autophagy to prevent the degradation of these abovementioned aberrant proteins, leading to their accumulation and deposition, and eventually to AD.^118^, ^119^ Further evidence indicates autophagy reduction occur in astrocytes and primary neurons infected by HHV‐6A, potentially due to altered lysosomal acidification.^119^

Interaction between autophagy and UPR activation is of high importance, since the latter may promote autophagy to alleviate endoplasmic reticulum (ER) stress in cells.^62^ Autophagy dysfunction can activate ER stress and contribute to triggering the UPR, resulting in an increase in Aβ production and a hyperphosphorylation of the tau protein in astrocytoma cells and primary neurons.^119^ However, the ER stress sensor protein kinase R‐like endoplasmic reticulum kinase (PERK) inhibitor GSK2606414 can reduce tau phosphorylation following HHV‐6A infection.^119^ Moreover, the UPR can activate glycogen synthase kinase‐3β (GSK‐3β), which is known to directly phosphorylate tau protein.^92^, ^120^ Another consequence of autophagy reduction and UPR activation caused by HHV‐6A infection is related to the activation of pro‐inflammatory pathways, including the STAT3, NF‐kB, and mTOR pathways, leading to the release of inflammatory cytokines such as IL‐6, IL‐1β, and CXCL13, further promoting neuroinflammation.^96^

HSV‐1 encodes the neurovirulence protein ICP34.5, which can interact with Beclin 1 and hinder the formation of autophagosomes by promoting the dephosphorylation of eIF2α. This inhibition of autophagy prevents the degradation of Aβ and contributes to AD pathology.^79^ However, lysosome‐associated membrane protein 2 (LAMP2) deficiency results in an apparent reduction in Aβ levels and tau hyperphosphorylation.^121^ Moreover, a hypothesis suggests that HSV‐1 infection induces Aβ peptide production in the brain by enhancing the endocytosis of APP, and these peptides are subsequently released from cells via exocytosis, thereby promoting AD development.^122^ In addition, the accumulation of Aβ in autophagic vesicles induced by HSV‐1 infection is caused by the inhibition of Aβ secretion and the failure of Aβ degradation by autophagy.^122^

Oxidative stress plays a pivotal role in causing direct damage to the brain, contributing to the pathogenesis and progression of AD.^123^, ^124^ Previous studies evaluated the interaction between oxidative stress and HSV‐1 infection in the appearance of neuropathological hallmarks of AD. Results indicated that oxidative stress could profoundly potentiate the accumulation of intracellular Aβ and inhibit its secretion, which is mediated by HSV‐1 infection.^125^ With respect to autophagy, oxidative stress induced by HSV‐1 infection triggered the accumulation of autophagosomes and inefficient fusion between autophagosomes and lysosomes.^125^ This oxidative stress‐mediated inhibition of autophagic flux could contribute to the increased accumulation of intracellular Aβ in autophagosomes in HSV‐1‐infected cells. Moreover, it has been demonstrated that oxidative stress and HSV‐1 infection led to an increase in the lysosome load and a reduction in the activity of lysosomal hydrolases.^126^ These findings suggest that alterations in the lysosomal system may contribute to the pathogenesis of AD.^126^ Additionally, oxidative stress and HSV‐1 infection also impede the activity of cathepsins, specifically cathepsin B, which is crucial in the degradation of key AD‐associated proteins, including Aβ, APP, and BACE1.^126^, ^127^

### 4.3 Other Neurodegenerative diseases

Viruses, such as HSV‐1/2, may be environmental factors contributing to ALS pathogenesis.^49^ Recently, in a mouse model infected with HSV‐2, the decreased protein level of chromosome 9 open reading frame 72 (C9ORF72) was highly associated with ALS pathogenesis.^47^, ^128^ A G4C2 repeat expansion in C9ORF72 is responsible for triggering the phosphorylation and aggregation of TBK1, leading to a loss of its activity to disrupt endosome maturation and induce TDP‐43 aggregation.^129^ Moreover, the genes involved in autophagy, such as SQSTM1/P62 or TBK1, are the critical defense mechanisms leveraged by neurons and glial cells to control HSV infections, which are also associated with ALS pathogenesis.^47^, ^130^ Recently, RNA sequencing performed on microglia from aged mice indicated that the most highly upregulated factors were CCL3, CCL4, and CCL5.^131^, ^132^ These factors could bind and activate neuronal CCR5, which in turn promotes mTORC1 activation by promoting the PI3K‐AKT‐TSC2 pathway and inhibiting autophagy.^132^, ^133^ However, the anti‐HIV drug, maraviroc, selectively blocked CCR5, which ameliorated Huntington's disease (HD) and tau pathologies in mouse HD models.^132^

## 5 GENE INTERACTION NETWORK OF HSV‐1 INFECTION, AUTOPHAGY AND NEURODEGENERATIVE DISEASES

Recent evidence supports the idea that a diverse range of viruses play pivotal roles in ND pathogenesis.^51^ Among these viruses, HSV‐1 is the most studied species in the family of neurotropic viruses. HSV‐1 infection can enter a state of latency, enabling it to persist indefinitely within the human host following infection. The reactivation of this virus from its latent state is particularly prevalent in elderly individuals and participates in the development of NDs.^122^ Here, we analyzed ARGs involved in HSV‐1 infection and ND pathogenesis and explored how these genes contributed to the onset and development of NDs during viral infection.

### 5.1 Autophagy‐related genes in HSV‐1 infection

**FIGURE 4:**
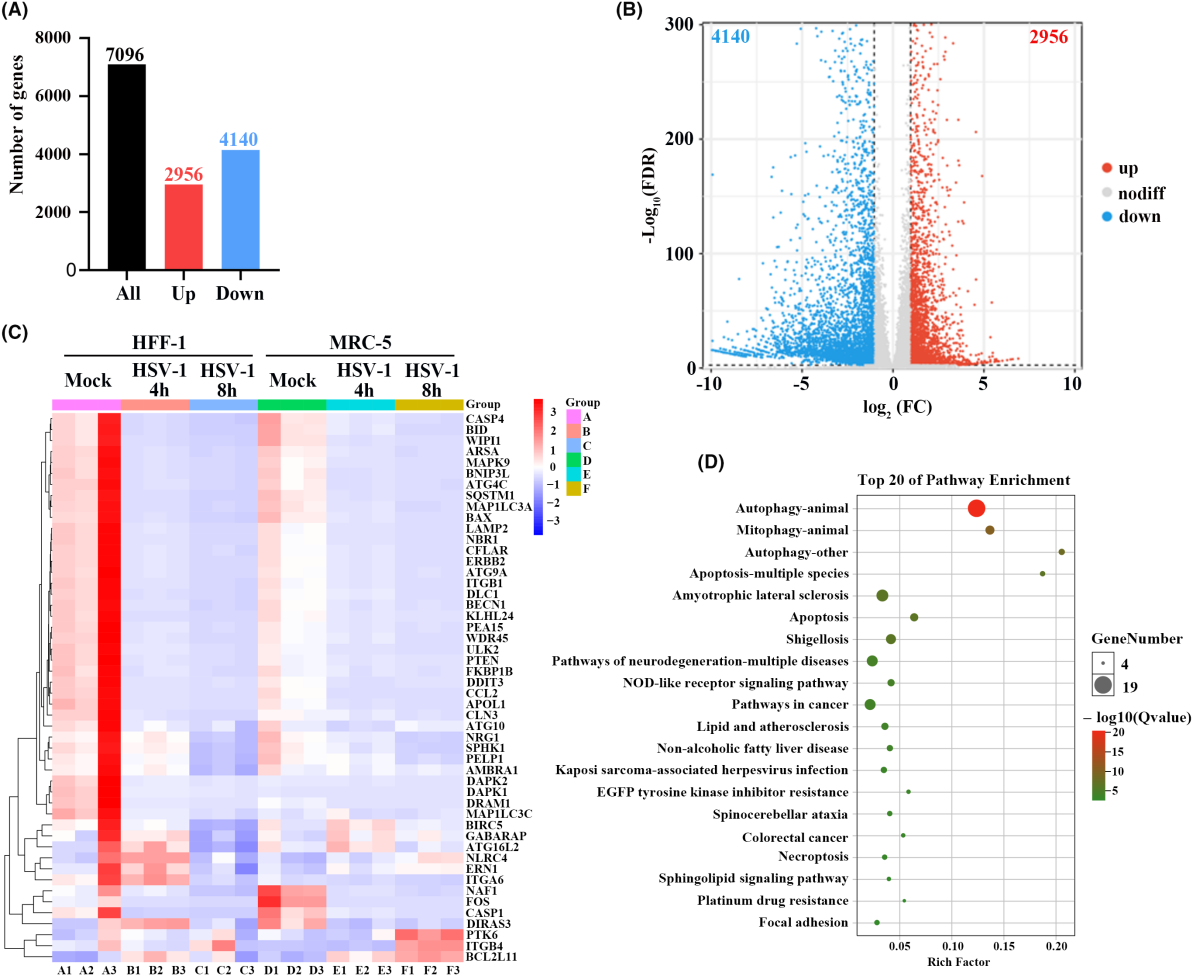
Differentially expressed genes (DEGs) in the HSV‐1‐infected cells from the SGE124118 dataset. (A, B) The numbers of up‐regulated and down‐regulated DEGs in the HSV‐1‐infected cells. (C) Intersection of DEGs and autophagy‐related genes upon HSV‐1 infection. (D) Bubble map of the top 20 most enriched signaling pathways.

To further investigate a potential correlation between autophagy and HSV‐1 infection, a total of 232 ARGs were extracted from the Human Autophagy Database (http://www.autophagy.lu/index.html). Differentially expressed genes (DEGs) in HSV‐1‐infected cells were identified using the GSE124118 dataset obtained from the NCBI Gene Expression Omnibus (GEO). DEGs were identified based on the criteria of FDR <0.05 and [log_2_ (fold change)] > 1, resulting in a total of 2956 up‐regulated and 4140 down‐regulated DEGs (Figure 4A,B). These gene sets were intersected, and 50 overlapping genes were ultimately obtained (Figure 4C). Among these genes, twenty‐five were found to be up‐regulated and 15 were down‐regulated. Subsequently, all these DEGs were mapped to the Kyoto Encyclopedia of Genes and Genomes (KEGG) database. Pathways with FDR‐corrected *p* values ≤0.05 were defined as significantly enriched pathways. A majority of the DEGs were found to be enriched in the autophagy pathway upon HSV‐1 infection (Figure 4D). Notably, endoplasmic reticulum to nucleus signaling 1 (ERN1), Beclin1, and sequestosome 1 (SQSTM1) were previously investigated to be associated with HSV‐1 infection.

ERN1 is a prominent factor involved in ER stress and has been demonstrated to induce autophagy during viral infection.^134^ Previous studies provided evidence that the ERN1/X‐Box binding protein 1 (XBP1) axis of the UPR was effectively suppressed upon HSV‐1 infection.^135^ Further investigation revealed that the viral protein UL41 could degrade XBP1, thereby inhibiting its expression.^136^ However, the activities of ERN1 RNase and kinase play distinct roles during viral infection with an increase in RNase activity and an inhibition in kinase activity.^137^ Beclin 1, a crucial protein involved in autophagy, regulates this process by interacting with Vps34 and other cofactors to form the Beclin 1 complex.^138^ Additionally, the serine/threonine kinase U3 of HSV‐1 directly phosphorylates ATG6/Beclin1, leading to the inhibition of its activity, which further suppresses cellular autophagy.^80^ Moreover, the HSV‐1 neurotoxic protein, ICP34.5, binds ATG6/Beclin1, resulting in the inhibition of its autophagic function.^79^ SQSTM1 serves as a mediator between ubiquitinated cargos and phagophores, facilitating the degradation of ubiquitinated cargos through autophagy.^139^, ^140^ Upon HSV‐1 infection, the expression level of SQSTM1/p62 was decreased at the early stages of viral infection.^141^ Moreover, the degradation of HSV‐1 VP16 was found to be associated with SQSTM1/p62‐mediated selective autophagy.^142^

### 5.2 Autophagy‐related genes in Neurodegenerative diseases

**FIGURE 5:**
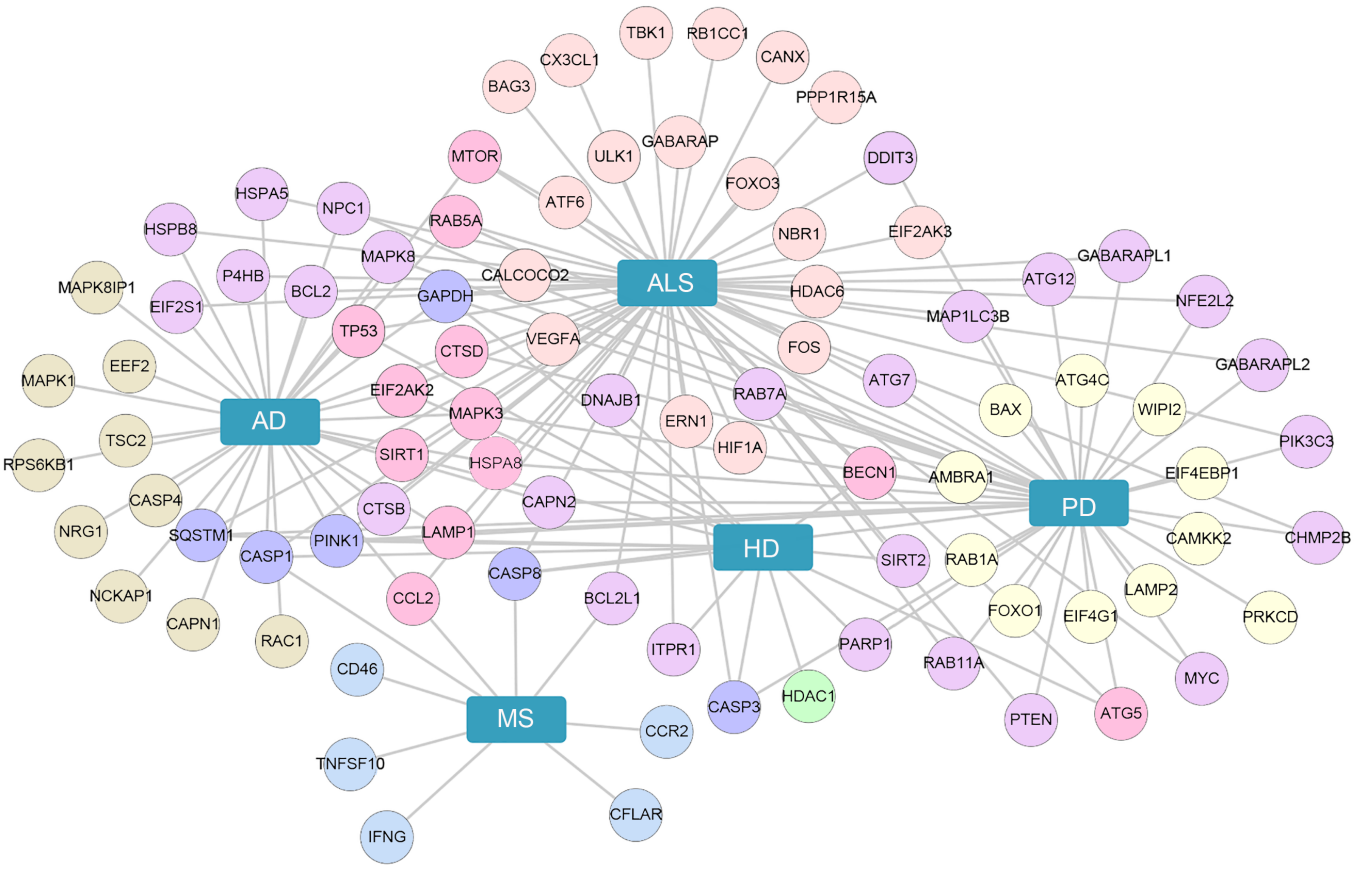
Autophagy‐related genes in NDs. Gene interaction network of autophagy‐related genes and host genes associated with NDs from MalaCards DB. The network was built using Cytoscape v.3.8.0.

Several genes associated with NDs have been discerned to regulate autophagy pathways, and mutations in these genes result in autophagy defects, which are believed to play a vital role in NDs pathogenesis.^143^ For instance, PTEN‐induced kinase 1 (PINK1) and Parkin were identified in patients with PD,^144^ while C9orf72, OPTN, and VCP were discovered in patients with ALS.^145^ Here, host genes associated with NDs from MalaCards DB (http://malacards.org/) were obtained. The intersection of these genes and ARGs showed that 41 ARGs were involved in PD, 34 in AD, 9 in MS, 16 in HD, and 59 in ALS. Notably, CASP3, GAPDH, PINK1, and SQSTM1 were closely associated with HD, ALS, AD, and PD (Figure 5).

PINK1 is confirmed to be involved in the process of mitophagy, which is a specialized form of autophagy.^146^ Defective mitophagy or PINK1 mutations were highly associated with NDs, including AD, PD, ALS, and HD.^147^ Previous studies indicated that PINK1 collaborated with PRKN, an E3 ubiquitin ligase, to target damaged mitochondria and deliver them to the lysosome for degradation.^144^, ^148^ Moreover, PINK1 can directly activate mitophagy by recruiting the autophagy receptors, OPTN, and nuclear dot protein 52 (NDP52), even in the absence of parkin.^149^ Moreover, mutations in PINK1 could disrupt the interaction between PINK 1 and Beclin1, resulting in the loss of its ability to enhance autophagy.^150^ However, recent studies revealed that PINK1 deficiency led to neurodegeneration by impacting protein phosphorylation rather than mitochondrial autophagy.^151^ This deficiency caused a notable decrease in the phosphorylation of various proteins crucial for neuronal survival without affecting mitochondrial morphology or dynamics.^151^, ^152^ In addition, PINK1 has also been implicated in the regulation of tau phosphorylation through the PI3K/Akt/GSK3β and Nrf2 signaling pathways in AD animal models.^153^

The selective autophagy receptor, SQSTM1, has been identified as being associated with NDs.^154^, ^155^ Defective autophagy mediated by SQSTM1 accelerated the aggregation of misfolded proteins, such as Aβ, tau, α‐syn, and huntingtin, which were implicated in AD, PD, and HD, respectively.^156^ In 2010, a mutation in huntingtin disrupted the recognition of cargo mediated by SQSTM1, resulting in failed autophagosome sequestration in HD cells.^157^ Moreover, huntingtin could interact with SQSTM1 to initiate the formation of autophagosomes, further facilitating the degradation of mutant huntingtin protein.^158^ Additionally, other host factors collaborated with SQSTM1 to regulate autophagy. For instance, the neighbor of the brca1 gene (NBr1) was found to impact ND pathogenesis via cooperatively modulating α‐syn aggregation.^159^ On the other hand, heat shock protein B1 (HSPB1) plays a crucial role in the formation of autophagosomes and can modulate autophagy through its interaction with SQSTM1.^160^ Subsequent investigations have demonstrated that the HSPB1‐SQSTM1 complex serves as a cargo‐loading platform, facilitating the noncanonical secretion of mutant huntingtin by extracellular vesicles.^161^ Besides, Bcl‐2 influences the affinity of p62 for poly‐ubiquitin chains, thereby suppressing the aggregation of poly‐ubiquitinated proteins, including mutant huntingtin.^162^

### 5.3 Cross‐talk among autophagy‐related genes during HSV‐1 infection and neurodegenerative pathogenesis

HSV‐1 infection and autophagy are associated with ND pathogenesis. HSV‐1 infection modulates autophagy through protein interactions between viral and autophagy‐related proteins or cellular signaling pathways.^79^, ^138^ Analysis of the GSE124118 dataset, which includes the transcriptomes of HSV‐1‐infected cells, revealed a decrease in the expression level of the selective autophagy receptor, SQSTM1, following viral infection, consistent with previous studies.^141^ Moreover, the mediated defective autophagy by SQSTM1 has also been implicated in the pathogenesis of NDs. These findings indicated that SQSTM1 served as a crucial gene linking HSV‐1 infection to NDs.

## 6 CONCLUSION

Overall, it is widely acknowledged that viral infections contribute as risk factors for NDs and the exact mechanisms underlying their involvement in ND pathogenesis remain incompletely comprehended. Autophagy can serve as the bridge between viral infection and NDs, as viral infectivity may be enhanced by modulating autophagy, which may predispose to NDs. Therefore, it is imperative to investigate and identify efficacious target sites that regulate autophagy, with a specific emphasis on NDs. These novel findings are likely to contribute to the advancement of new therapeutic strategies not only for NDs but also for viral infections. Currently, there has been evidence suggesting that antiviral agents might potentially mitigate ND progression, but this remains a future goal for those involved in drug development and repurposing efforts.

## AUTHOR CONTRIBUTIONS

ZYJ and ZF designed the review. ZYJ drafted the manuscript. XKF designed the figures and table. SFX analyzed the GEO data. ZF revised the review. Both authors approved the final version of the manuscript for submission.

## CONFLICT OF INTEREST STATEMENT

All authors declare no financial or non‐financial competing interests.

## CONSENT TO PUBLISH

All authors consent on publication of this review article.

## Data Availability

All data generated or analyzed during this study are included in this published article.
